# Impact Melt Facies in the Moon's Crisium Basin: Identifying, Characterizing, and Future Radiogenic Dating

**DOI:** 10.1029/2019JE006024

**Published:** 2020-01-05

**Authors:** K. D. Runyon, D. P. Moriarty, B. W. Denevi, B. T. Greenhagen, G. Morgan, K. E. Young, B. A. Cohen, C. H. van der Bogert, H. Hiesinger, L. M. Jozwiak

**Affiliations:** ^1^ The Johns Hopkins University Applied Physics Laboratory Laurel MD USA; ^2^ NASA Goddard Space Flight Center Greenbelt MD USA; ^3^ Planetary Science Institute Tucson AZ USA; ^4^ Institut für Planetologie University of Münster Münster Germany

**Keywords:** dating, lunar, cataclysm, geochronology, cratering, Moon

## Abstract

Both Earth and the Moon share a common history regarding the epoch of large basin formation, though only the lunar geologic record preserves any appreciable record of this Late Heavy Bombardment. The emergence of Earth's first life is approximately contemporaneous with the Late Heavy Bombardment; understanding the latter informs the environmental conditions of the former, which are likely necessary to constrain the mechanisms of abiogenesis. While the relative formation time of most of the Moon's large basins is known, the absolute timing is not. The timing of Crisium Basin's formation is one of many important events that must be constrained and would require identifying and dating impact melt formed in the Crisium event. To inform a future lunar sample dating mission, we thus characterized possible outcrops of impact melt. We determined that several mare lava‐embayed kipukas could contain impact melt, though the rim and central peaks of the partially lava‐flooded Yerkes Crater likely contain the most pure and intact Crisium impact melt. It is here where future robotic and/or human missions could confidently add a key missing piece to the puzzle of the combined issues of early Earth‐Moon bombardment and the emergence of life.

## Key Points

- Relatively pure impact melt from Crisium Basin is exposed in the central peaks of Yerkes Crater
- Diluted Crisium impact melt may be exposed in some previously identified kipukas in Crisium
- Radiogenic dating of impact melt exposed in Yerkes' central peak during a future mission would yield the age of the Crisium impact event

## 1 Introduction

### 1.1 Background and Motivation

Understanding the first billion years (~4.5–3.5 Ga) of the impact history of the inner solar system has profound implications for deciphering the early evolution of planets, the influence basin formation has on surface development, and, crucially, testing the Terminal Lunar Cataclysm (TLC) hypothesis (e.g., Tera et al., 1974). The hypothetical TLC is a spike in the lunar impactor flux from ~4–3.8 Ga proposed to explain the clustering of radiogenic ages of lunar samples around 3.9 Ga (e.g., Kring & Cohen, 2002; Michael et al., 2018; Morbidelli et al., 2012; Tera et al., 1974). The TLC would require that most of the Moon's basins and large craters formed within a timespan of only ~200 Myr. We use the term TLC to describe this proposed spike in impactor flux, whereas the more general term Late Heavy Bombardment implies higher past impactor flux, but not necessarily concentrated in a spike, following the taxonomy of Morbidelli et al. (2018).

Knowing the magnitude and duration of the Late Heavy Bombardment helps constrain the environmental boundary conditions of the approximately contemporaneous emergence and evolution of Earth's first life (Cohen et al., 2018; Mojzsis et al., 1996). On Earth, the surface and near‐subsurface expression of the Hadean‐ and earliest Archean‐aged crust has long been destroyed or severely metamorphosed (Trail et al., 2007, and references therein; Abramov & Mojzsis, 2009). In contrast, the relative quiescence of the Moon's geology resulted in the preservation of ancient impact structures. The Moon's early impactor flux is, thus, the best available proxy both for understanding Earth's early impactor flux (Cohen et al., 2000; Hartmann, 1975; Kring & Cohen, 2002; Ryder, 1990; Tera et al., 1974; Kring, 2003), thereby constraining the environmental boundary conditions at the time of life's emergence on Earth ~3.8 Ga (Mojzsis et al., 1996).

The TLC hypothesis has long been controversial, and conflicting data and interpretations exist (e.g., Hartmann, 1975; Michael et al., 2018; Ryder, 1990). For example, recent analyses of Apollo 14–17, Luna 20, and lunar meteorite ^40^Ar‐^39^Ar ages have been interpreted to show a generally elevated impactor flux from 4.25–3.87 Ga rather than a spike from 4–3.8 Ga (Michael et al., 2018). Others have suggested that any apparent spike in impactor flux could be explained if the dated samples primarily originated in a single, large basin: Imbrium (Cadogan et al., 1977; Cohen et al., 2018; Haskin, 1998; Norman et al., 2010; Stettler & Albarede, 1978; Swindle et al., 1991; Van der Bogert et al., 2018).

We thus turn our attention to understanding the timing of the formation of key lunar impact basins. Relative ages can be interpreted from superposition relationships (Shoemaker & Hackman, 1962; Wilhelms & McCauley, 1971; Spudis et al., 2011; Wilhelms, 1984) and superposing crater size‐frequency distributions, which can also be used to estimate absolute ages via the lunar cratering chronology function (e.g., Fassett, 2016; Hartmann, 1970; Hiesinger et al., 2012; Neukum, 1983; Neukum et al., 1975). For impact events, absolute ages must come from radioisotope dating of basin impact melt lithologies either from returned samples in Earth‐based laboratories or in situ on the Moon (e.g., Cohen et al., 2018). However, unambiguous identification of samples from basins other than Imbrium is difficult using the Apollo and Luna collection (Cohen et al., 2018). Because of their superposition relationships, better constraints on the formation time of several key basins, namely, the lunar nearside basins Nectaris, Serenatatis, and Crisium, can help better define the timing of the nearside basin epoch—whether there was a sustained period of formation or a sudden increase in impactor flux (Spudis et al., 2011; Spudis & Sliz, 2017).

Here, we focus on the Crisium basin with the goal of identifying exposures of impact melt from which radiogenic ages could provide an unambiguous date of the Crisium impact event. We examine the morphology and morphometry, topography, reflectance, composition, and contextual geology of deposits mapped as Crisium impact melt by Spudis and Sliz (2017) in order to ascertain which are the most likely to have a high concentration of melt with the least amount of mixing and subsequent impact processing.

### 1.2 The Crisium Basin

#### 1.2.1 General Geology

**Figure 1:**
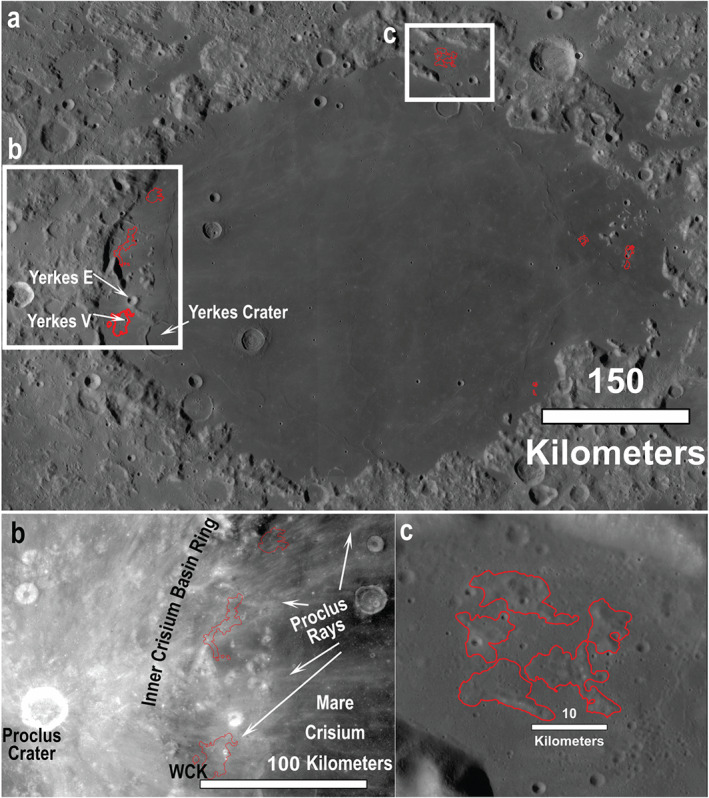
(a) A general overview of Mare Crisium (100‐m WAC global mosaic) showing the remapped kipukas outlined in red (after Spudis and Sliz, 2017) located near the peak ring complex. (b) Low incidence angle (“high Sun”) WAC mosaic of the Western Crisium Kipuka (WCK) area and the widespread feldspathic highlands contamination from Proclus Crater's ejecta rays. (c) Medium incidence angle WAC mosaic of the northern Crisium Archipelago of kipukas. The WCK and Northern Archipelago are shown in greater detail in Figures 2 and 3. Base image credit: NASA/GSFC/Arizona State University.

Mare Crisium (~400 × 580 km) is the lava‐flooded portion of the Nectarian‐aged Crisium Basin (Spudis et al., 2011; *D* ~ 1,000 km) and is centered near 17.0°N, 58.8°E (Figure 1), isolated from other maria and basins by highlands terrain. It is ringed by highland massifs that form the basin's innermost and best preserved rings, interpreted to be structurally equivalent to Orientale's Inner Rook Ring (Spudis & Sliz, 2017). The basin is surrounded by feldspathic highlands and is far removed (>900 km) from the Procellarum KREEP Terrane (Wieczorek & Phillips, 2000). Bouguer gravity analysis from the Gravity Recovery and Interior Laboratory (Zuber et al., 2013) mission indicates that the Crisium impact may have removed all of the crust in portions of the basin, resulting in a current crustal thickness of ~0 km (Wieczorek et al., 2013). Thus, we expect Crisium impact melt to contain a significant lower crustal and possibly mantle component, and therefore a relatively mafic mineralogy.

#### 1.2.2 Crisium Basin Impact Melt Extent and Thickness

Estimates of the extent and thickness of Crisium's central impact melt sheet can be made through comparisons with the Orientale basin (~920 km in diameter), where flooding by mare basalts was less extensive and thus impact melt facies are more readily observed (e.g., Head*,* 1974). The 364‐km‐diameter (Neumann et al., 2015) inner depression or “bench” within Orientale (interior to the Inner Rook Ring) has been suggested to result from the impact excavation, downward target displacement, and subsequent subsidence of the impact melt sheet due to volume changes during melt cooling and crystallization (Bratt et al., 1985; Wilson & Head, 2011; Vaugan et al., 2013). Based on the magnitude of subsidence, Vaugan et al. (2013) estimate the Orientale melt sheet to be ~14 km thick in the deepest and most central regions. Gravity measurements from Gravity Recovery and Interior Laboratory suggest in a thinner maximum estimate of ~10–11 km (Zuber et al., 2016), and stratigraphic relationships suggest that the melt sheet thins to ~6 km near the inner Rook ring (Spudis et al., 2014).

An inner depression (bench) analogous to that of Orientale is observed within Crisium (Zuber et al., 2016) and is defined by circumferential wrinkle ridges. The central depression within Crisium is ~380 km in diameter, ~5% wider than that of Orientale; the similar extent suggests comparable impact melt production and evolution. In fact, given the somewhat larger diameter of Crisium and the associated higher impact energy, the volume of impact melt produced by Orientale is well suited as a lower limit for that of Crisium. Therefore, it is likely that Crisium is host to an impact melt sheet ~10–15 km thick toward the middle and thinning to perhaps ~6 km toward the ring massifs (Spudis et al., 2014). It is likely that significant volumes of impact melt, breccia, and ejecta were emplaced in the surrounding zones, extending beyond the inner massif ring in a surface melt flow (Osinski et al., 2011) or “splash” consistent with observations of melt facies beyond Orientale's Inner Rook Ring (Spudis et al., 2014).

Yerkes Crater (diameter of 35 km) sits just outside the inner depression in southwest Crisium and is partially flooded by mare basalts with only the rim and central peak complex cropping out. Combining information from these multiple studies, we reason that the central peak, having been uplifted from a minimum depth of ~3.8–4 km (based on Cintala & Grieve, 1998: minimum uplift depth = 0.022*D* ^1.45^, where *D* is the final crater diameter), likely samples the middepths of the 6‐ to 15‐km‐thick Crisium impact melt sheet (Spudis et al., 2014; Vaugan et al., 2013; Zuber et al., 2016). Yerkes Crater is thus conveniently analogous to Maunder Crater (diameter of 55 km) with regard to size (35‐ vs. 55‐km diameter), location within its host basin (edge of Crisium/Orientale central depression), and excavation of basin impact melt (~4‐ vs. ~7‐km depth).

#### 1.2.3 Putative Exposures of Crisium Impact Melt

Spudis and Sliz (2017) identified kipukas (high‐standing terrain embayed on all sides by mare basalt) around the periphery of Mare Crisium as the possible remains of a buried melt sheet (Figure. 1). They based their impact melt interpretation on the resemblance of these deposits to some or all of the characteristics of the Orientale impact melt characteristics: (1) location near the basin's inner ring; (2) their expectation that melt should have a feldspathic highlands, nonmare composition; (3) older relative age than the mare; and (4) hummocky and/or fractured texture. Here, we reexamine and further characterize these kipukas of putative impact melt in addition to other potential impact melt outcrops—such as crater central peaks and rims—to test the hypothesis that they are impact melt from Crisium.

**Figure 2:**
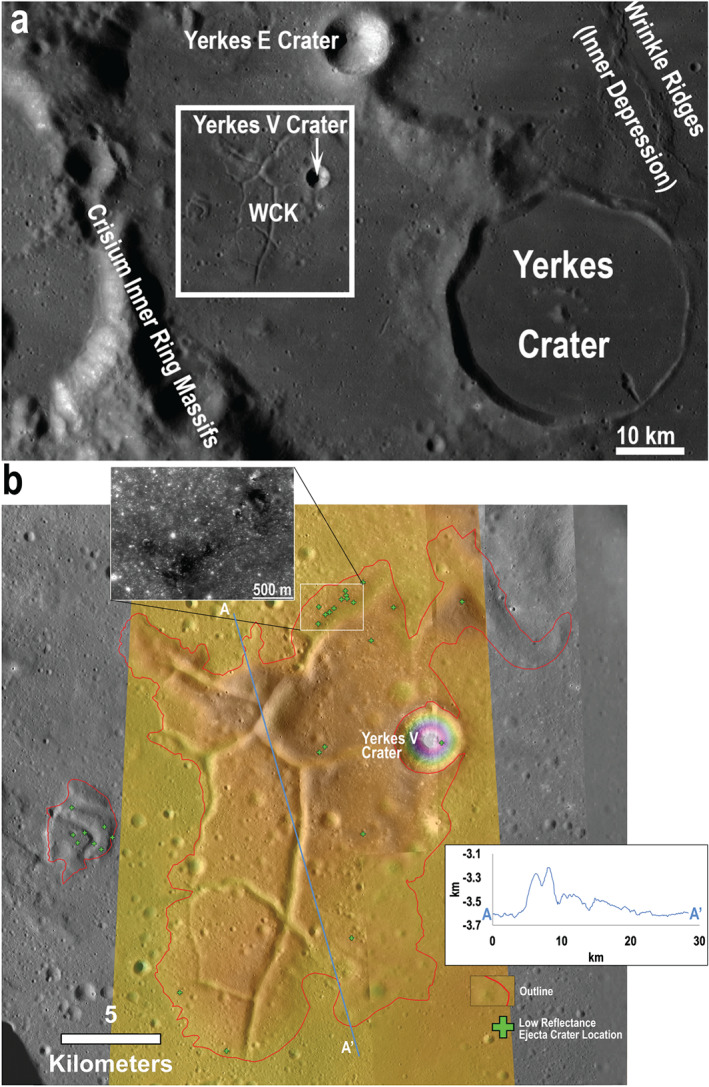
(a) Context including Yerkes Crater in western Crisium with the area in B shown by the white rectangle. Base image: LROC WAC mosaic from QuickMap/Arizona State University. (b) The 1:50,000 mapping of the Western Crisium Kipuka (WCK) with a colorized NAC DEM overlaid. Embayment and other topography differences define the mapped contact with a ±100 m uncertainty buffer. Low reflectance ejecta craters are mapped with green crosses, and are possibly indicative of excavated intrusive materials. Inset: The northernmost crater ejecta (green arrows) with low reflectance shows a strong reflectance contrast between the surface and subsurface material. The DEM shows a maximum of ~1 km of relief, from −3,202 m below lunar datum to −4202 m. Base image and DEM credit: NASA/GSFC/Arizona State University.

**Figure 3:**
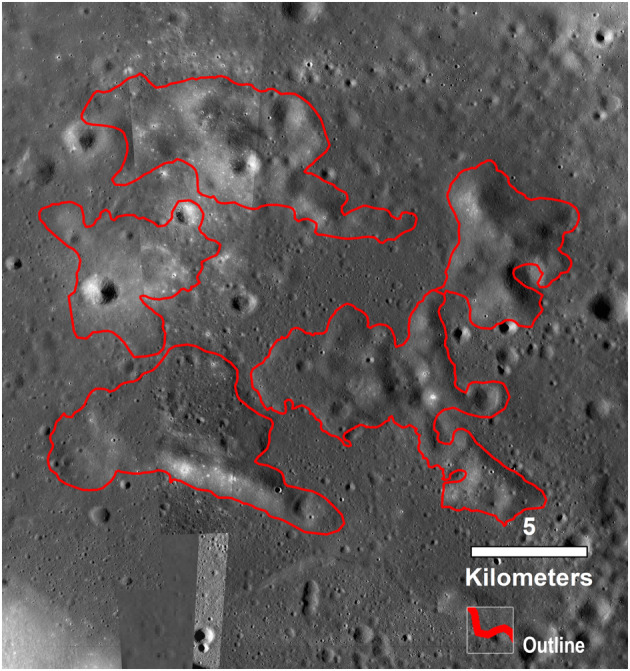
The 1:50,000 mapping of the Northern Crisium Archipelago. Base image credit: NASA/GSFC/Arizona State University.

While we examined and evaluated each of the previously identified sites, the geology of two regions is presented in detail. The two example regions are the Western Crisium Kipuka region (WCK; referred to as Remnant A in Van der Bogert et al., 2018) near Yerkes Crater (Figure 2); and the Northern Crisium “Archipelago” of five kipukas (Figure 3). The kipukas at each site rise tens to hundreds of meters above the embaying mare flood basalts, span tens of kilometers in lateral extent, and are located near the ring massifs.

To the west of the WCK, outside Mare Crisium, the fresh crater Proclus (*D* ~ 27 km) features high‐reflectance rays that extend over much of western Mare Crisium, including the WCK. The WCK features a 3.5‐km crater, Yerkes V, on its northeast boundary. The rim of Yerkes V Crater is 22 km from the rim of Yerkes Crater (Figure 2). Toward the basin's center from the WCK is a circumferential wrinkle ridge interpreted, based on analogy to Orientale, to be the surface manifestation of the basin's thickest melt sheet deposit, sometimes called an inner depression (Figure 2; e.g., Baker et al., 2016; Zuber et al., 2016; Ji et al., 2018).

The Archipelago sits between a bifurcated arm of Crisium's innermost massif ring and is surrounded by more degraded craters than the WCK. The 42‐km‐diameter crater Eimmart sits 67 km to the east. Eimmart C Crater sits 22 km to the south and appears floor‐fractured, mareflooded, and is cut by a wrinkle ridge. Eimmart F Crater (diameter of 8 km) is the freshest large crater near the Archipelago.

## 2 Methods

### 2.1 Data Sets

High‐resolution Lunar Reconnaissance Orbiter Camera (LROC) Narrow Angle Camera (NAC) images with pixel scales at or below 2 m (Robinson et al., 2010) form the basis of our mapping (Table S1). The high resolution of these images allows for high‐fidelity identification of the geologic contacts between the kipukas and the surrounding mare. LROC Wide Angle Camera (WAC) images (100 m per pixel) provide contextual views of the surrounding landscape. NAC images with low incidence angles (Sun high in the sky; no shadows) and high incidence angles (Sun low in the sky; long shadows) provide complementary views of each scene, where the former is well suited for measuring reflectance differences and the latter for discerning geomorphology.

We used the U.S. Geological Survey Integrated Software for Imagers and Spectrometers (Keszthelyi et al., 2013) program “lronacpho” to calculate photometrically normalized reflectance from low incidence angle NAC images of the WCK, Archipelago, and other Crisium areas for comparison. The images were normalized to 30° incidence angle, 0° emission angle, and 30° phase angle using an empirical model from Boyd and Robinson (2018) with photometric parameters of A0 = –2.649, A1 = –0.013, A2 = –0.274, and A3 = 0.965.

Stereo imaging from LROC provides both global (WAC; 100‐m ground sample distance; Scholten et al., 2012) and local (NAC; 2‐ to 6‐m ground sample distance; Henricksen et al., 2015) digital elevation model (DEM) topographic coverage. A DEM from the combined Lunar Orbiter Laser Altimeter (LOLA) and stereoscopic Kaguya Terrain Camera provides a global 59‐m ground sample distance (Barker et al., 2016). DEMs allow measurement of topographic profiles for extraction of, for example, ground slopes. While the NAC DEMs provide extremely high‐resolution topographic information, they are few in number and not always available for a landform of interest, so we supplemented measurements with the WAC DEM. We found that slopes calculated from the WAC DEM agreed with those calculated from a NAC DEM when a given lunar feature spanned at least five sample points in the lower resolution WAC data. We extracted profiles from the DEMs in ArcMap and evaluated the topography data in MATLAB and Excel.

### 2.2 ArcGIS Mapping of Kipukas

Using the mapped kipukas identified by Spudis and Sliz (2017) as a starting point, we remapped the same areas at a scale of 1:50,000 using LROC NAC images acquired at moderate incidence angles (some shadows present), and with context provided by the 100‐m WAC mosaic (Figures 2 and 3). While Spudis and Sliz (2017) did not report a mapping scale, their work was limited to the 100 m/pixel WAC mosaic. Slopes derived from the DEMs (NAC, WAC, and LOLA/Terrain Camera) provided an additional basis for the interpretation of geologic contacts when used in conjunction with NAC images; a NAC DEM was available only for the WCK.

We followed the morphological mapping criteria of Spudis and Sliz (2017) and Sliz and Spudis (2016) and identified the contact between the kipukas and mare based primarily on the contrast in texture between the smooth, lightly cratered mare and the rough, knobby, and sometimes fractured kipukas. The uncertainty in the position of the kipuka‐mare contact is up to 100 m in some regions due to locally ambiguous mare embayment.

### 2.3 Spectral Analysis

**Figure 4:**
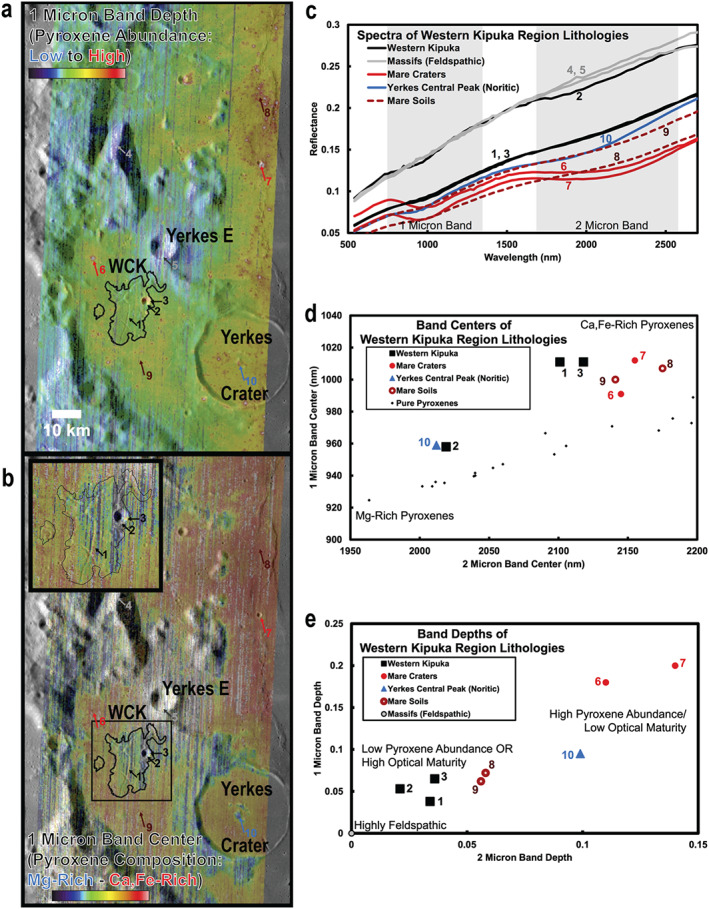
(a) Parameter map of the 1‐μm band depth, derived from M^3^ image M3G20090604T191631 using the Parabolas and two‐part Linear Continua (PLC) technique developed and validated by Moriarty and Pieters (2016). This parameter is sensitive primarily to the abundance of mafic minerals (which on the lunar surface is predominantly pyroxene). The parameter is also sensitive to optical maturity, as optically mature materials exhibit weaker spectral absorptions than optically immature materials of the same mineralogy. (b) Parameter map of the 1‐μm band center, derived from the same M^3^ image, also using the PLC technique. This parameter is sensitive to pyroxene composition, as Mg‐rich pyroxenes exhibit relatively short‐wavelength absorption bands, while Ca,Fe‐rich pyroxenes exhibit longer‐wavelength absorption bands. Together, these parameter maps characterize the compositional diversity of the region. Inset: detail of the Western Crisium Kipuka. (c) Spectra from the Western Crisium Kipuka compared to representative spectra from diverse materials across the region. Spectra are 3 × 3 pixel averages from M^3^ image M3G20090604T191631, with the exception of the Mare Soils spectra, which are 10 × 10 pixel averages. Spectra locations are indicated in (A + B). The 1‐ and 2‐μm band regions are shaded gray. (d) The band centers of the 1‐ and 2‐μm absorption bands derived from the spectra presented in (c) using the single‐spectrum PLC analysis. The band centers of Crisium materials are compared to band centers of synthetic pure pyroxenes analyzed by Klima et al. (2007, 2011) and measured using PLC (Moriarty & Pieters, 2016) to provide context for compositional interpretations, as band centers are sensitive to pyroxene composition. As discussed by Moriarty and Pieters (2016), natural materials often exhibit an offset from the synthetic pure pyroxene trend due to several factors including nonlinear mixing of several pyroxene and nonpyroxene components, natural crystallization processes such as zoning and exsolution, and space weathering. (e) The band depths of the 1 and 2‐μm absorption bands of these spectra derived using the same technique. Band depths are sensitive to pyroxene abundance and optical maturity. See text for results and discussion.

**Figure 5:**
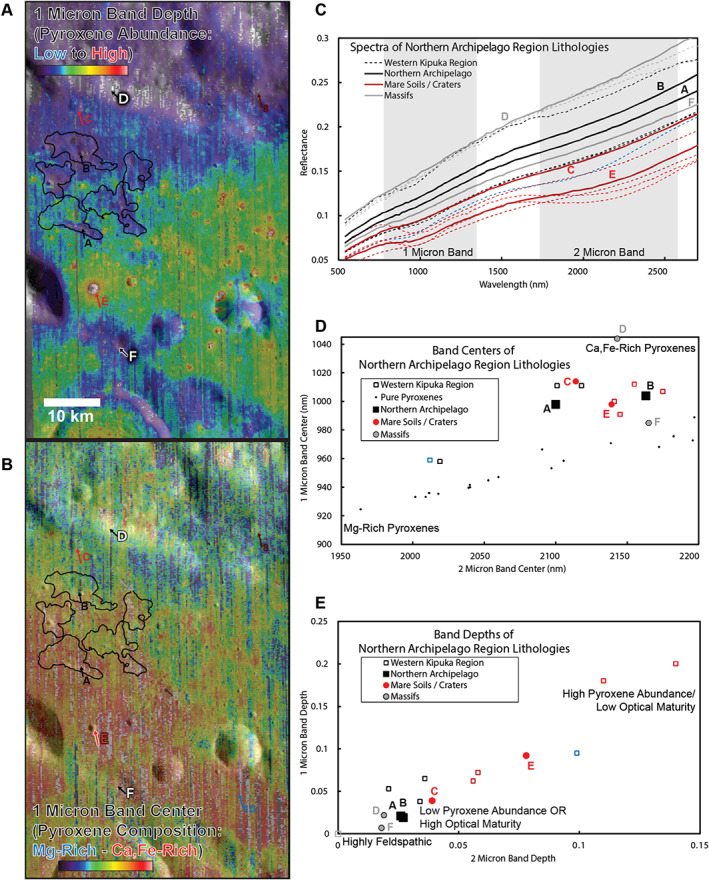
Same sense as Figure 4 but for the Northern Archipelago of kipukas. See text for results and discussion.

We used visible‐near‐infrared reflectance spectra from the Moon Mineralogy Mapper (M^3^) (Pieters et al., 2009) to evaluate the mineralogy of the mapped kipukas in comparison with their surroundings (Figures 4 and 5). For each region of interest, we analyzed one M^3^ image: M3G20090604T191631 for the WCK and Yerkes Crater, and M3G20090603T221232 for the Northern Archipelago. M^3^ operated under several different optical periods, which affected behavior of the instrument. To enable a direct comparison of the areas of interest, these images are both from Optical Period OP2C1 (Besse et al., 2013). The M^3^ images are from the Level 2 (L2) reflectance data available on the PDS. The L2 data includes a thermal and photometric correction to standard viewing geometry. An additional ground‐truth correction was applied (Isaacson et al., 2013).

Near‐IR spectral diversity of the lunar surface is dominated by the abundance and composition of pyroxenes, which exhibit diagnostic absorption bands at 1 and 2 μm. To evaluate mineralogical diversity across the region, we created parameter maps for these absorption bands using the Parabolas and two‐part Linear Continuum technique (PLC; Moriarty & Pieters, 2016). We created two sets of parameter maps: estimated band depth, sensitive to pyroxene abundance (high = mafic; low = feldspathic), and estimated band center, sensitive to pyroxene composition (short wavelengths = Mg rich; long wavelengths = Ca,Fe rich). These maps provide an overview of the mineralogical properties of the region and help identify areas for more detailed study. Spectra collected are 3 × 3 pixel averages (for fresh craters and outcrops) or 10 × 10 pixel averages (for soils) to increase the signal‐to‐noise ratio.

### 2.4 Christiansen Feature Analysis

**Figure 6:**
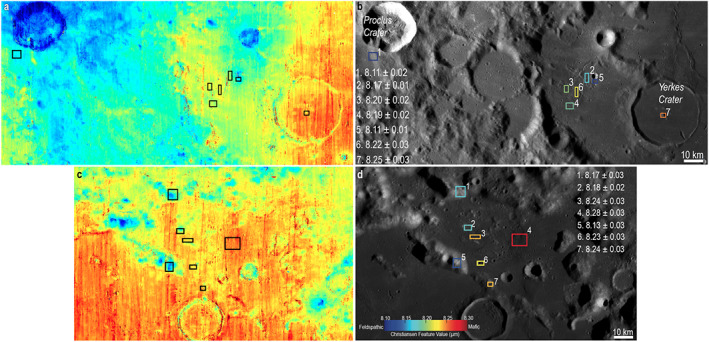
Center of the Christiansen feature for lithologies local to western Crisium (a and b) and northern Crisium (c and d). Higher numbers (warmer colors) are more mafic than cooler colors (more feldspathic). Notably, the central peak of Yerkes Crater has a more mafic Christiansen feature than anywhere on the WCK or Proclus Crater. The right column shows an interpreted representation of the Christiansen feature data in the left column for specific regions of interest. Image base maps: LROC WAC, NASA/GSFC/Arizona State University.

Fundamental molecular vibrations for silicate minerals occur in the thermal infrared between approximately 7.5 and 10 μm and give rise to diagnostic spectral features, including the Reststrahlen Bands, transparency features, and the Christiansen feature (CF; e.g., Salisbury & Walter, 1989). Spectroscopic thermal emission observation of the Moon's surface therefore provides an independent constraint on silicate composition that is complimentary to those from the visible and near‐infrared (e.g., Donaldson Hanna et al., 2014). We used data from the Diviner Lunar Radiometer (Diviner), a nine‐channel radiometer onboard the Lunar Reconnaissance Orbiter (Paige et al., 2010), to determine the position of the CF (the midinfrared emissivity maximum) and to discriminate between lithologies within the Crisium Basin. From the Diviner PDS‐archived Reduced Data Record data set, we selected rectangular regions of interest containing at least 100 radiogenic measurements taken with emission angles less than 2° off nadir and local solar times between 9:00 and 15:00. Using the method of Greenhagen et al. (2010, 2011), we normalized the data to equatorial noon, fit the Diviner Channels 3, 4, and 5 (7.8, 8.28, and 8.55 μm) using a second‐order polynomial, and interpolated the result to find the wavelength of maximum emission (i.e., the Christiansen Value). We then averaged the Christiansen Values and reported the mean and standard deviation for each region of interest (Figure 6).

### 2.5 Radar

**Figure 7:**
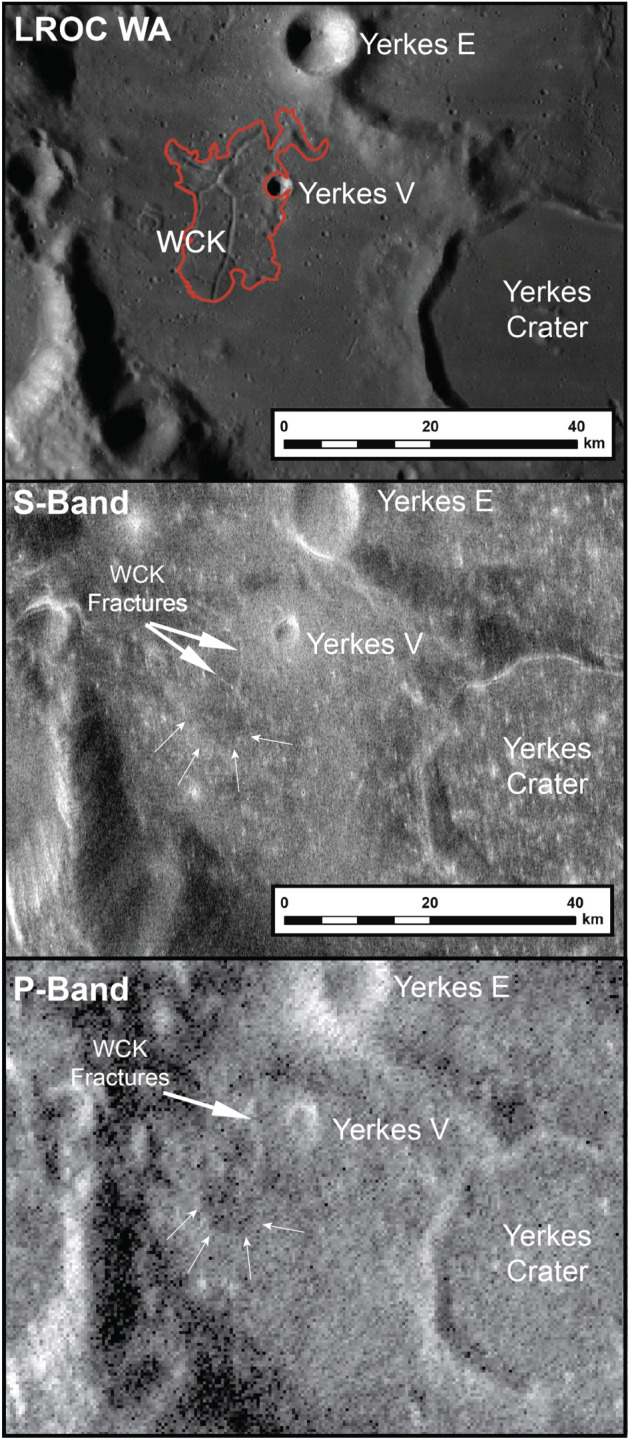
LROC WAC and corresponding Earth‐based P‐band SC coverage of the WCK. The radar data reveal multiple features not apparent in the image data, such as potential pyroclastic deposits and the possible continuation of fractures into the subsurface.

Earth‐based, S‐band (12.6‐cm wavelength) and P‐band (70‐cm wavelength) radar data were also applied to assist our geologic assessment of the kipukas (Figure 7). Radar backscatter provides information regarding the Fresnel reflectivity, signal attenuation (both a function of composition), surface roughness, and rock distributions. The ability of radar to probe beneath the surface (S‐band: <5 m; P‐band: <20 m) provides a unique perspective of the local geology relative to the image and spectral data sets described above. All of the radar data sets were obtained by using the Arecibo Observatory in Puerto Rico as a transmitter (of a circular‐polarized signal) and the Green Bank Telescope in West Virginia as a receiver (Campbell et al., 2007, 2010). Through this bistatic configuration, both senses of circular polarization were recorded. Both same sense circular (SC) polarization (relative to that transmitted) and circular polarization ratio (CPR) images were applied to this study. SC images are sensitive to diffuse scattering by rocks (S‐band: >1 cm; P‐band: >10 cm in diameter) at the surface and buried within the probing depth of the radar signal. CPR is the ratio of same sense to opposite sense polarization channels. High values are representative of blocky material within the probing depth and low values indicate rock‐poor material/mantles. The P‐band and S‐band images have pixel scales of 400 and 80 m, respectively.

## 3 Results

### 3.1 Refined Mapping of Kipukas Using LROC NAC Images

The boundaries of the kipukas are defined by mare‐kipuka embayment relations, which are typically detected on the basis of a distinct visual change in albedo and morphology (Figures 2 and 3). The ~1‐ to 2‐m pixel scale of the NAC images versus the 100 m per pixel WAC mosaics provided refinements in the determination of the margins and locations of kipukas relative to the original work of Spudis and Sliz (2017). We split a few areas previously mapped as one unit, and added more detail to the periphery of the kipukas. One region in eastern Crisium mapped by Spudis and Sliz was determined to be a field of secondary craters and not a kipuka (Van der Bogert et al., 2018); it was not included here. Results for our two regions of interest (section 1.2.3) are described below.

The WCK is ~29 km long and 18 km across at its widest point with an overall fractured dome morphology (Figure 2). It has an older absolute model age (3.94 Ga) than the surrounding mare (3.47 Ga; Van der Bogert et al., 2018). Yerkes V Crater (*D* = 3.5 km) straddles the contact between the WCK and the mare. A smaller kipuka, located 4 km to the west, features a similar domed and fractured morphology, though it is too small (~4 × 3 km) to derive meaningful crater statistics.

We mapped five individual kipukas that comprise the northern Crisium Archipelago, shown in Figure 3. While the Northern Archipelago lacks fractures, its terrain is rough and knobby, similar to impact melt in Orientale (Spudis & Sliz, 2017). The Archipelago measured ~18 × 18 km in its approximately NS and approximately EW dimensions. While we mapped the eastern kipukas in the Archipelago as two distinct deposits, ambiguous embayment relationships leave open the possibility that they are connected.

### 3.2 Fractures

**Figure 8:**
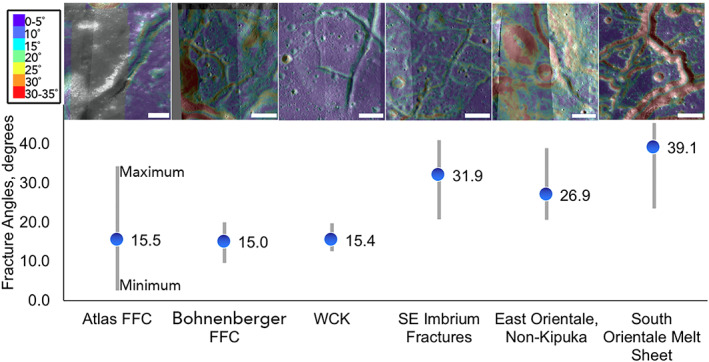
Comparison of qualitative geomorphology (images, top) and slopes from DEMs (plot, bottom) for floor‐fractured craters, the WCK fractures, Imbrium fractures (likely melt sheet), and the Orientale melt sheet fractures. The extents of the vertical gray bars indicate the range (minimum to maximum) measured. Within the minimum‐maximum range, floor‐fractured craters and the WCK have identical slope averages (blue dots) of ~15°. The Orientale melt sheet fractures and the Imbrium fractures have much higher slopes, notably beyond the ~33–34° angle of repose for many granular media. These higher slopes are consistent with a normal fault interpretation (Sorkhabi, 2012a, 2012b). All scale bars are 2 km. Image Credit: NASA/ASU. DTM Credit: NASA/LROC, JAXA/SELENE. Fracture measurement data in Table S2.

Spudis and Sliz (2017) used fractures as one criterion to help identify impact melt deposits. However, fractures are not unique to impact melt deposits on the Moon and therefore should be used with caution when identifying potential impact melt facies. Floor‐fractured craters (Jozwiak et al., 2012, 2015, 2017; Schultz, 1976) also feature ubiquitous fracturing on the same scale as impact melt fractures, though they are thought to form due to extensional faulting (Jozwiak et al., 2015) that results from uplift caused by a magmatic plug (Jozwiak et al., 2017). Impact melt, conversely, is thought to fracture from the flow of molten rock beneath a ductile crust (Bray et al., 2010) and contractions during cooling (Xiao et al., 2014). We measured the slopes found on fractures within the impact melt of Orientale, within floor‐fractured craters, and in the WCK as a possible means of discriminating between possible fracture origins (Figure 8). While it is unlikely that the slopes of fractures are uniquely related to their mode of formation, the measurements may reveal consistency among a single type.

Atlas and Bohnenberger craters, which are floor‐fractured craters, provide clear evidence for fracturing in response to floor uplift, and the morphology and morphometry of this process is representative of the processes modifying such craters. They can therefore be seen as points of comparison for investigating the fracturing mechanism of other locations on the Moon. These craters have average floor fracture slopes of 16° ± 8° (±1 standard deviation) and 15° ± 5°, respectively, which is low compared to typical normal faults, which often dip >30° (Sorkhabi, 2012a, 2012b). Measured fractures in the WCK have slopes of 15° ± 3°, indistinguishable from the slopes of fractures in the reference floor‐fractured craters. In contrast, the larger fractures of Orientale impact melt deposits (e.g., ~2 km across) have steeply sloping walls: large, kilometer‐scale fractures in the Maunder Formation have slopes of 39° ± 8°, with measured slopes as large as 45°. Similarly, slightly smaller fractures (~1 km across) in eastern Orientale impact melt deposits have average slopes of 27 ± 7°; though lower, these measurements are not inconsistent with a normal fault interpretation (Sorkhabi, 2012a, 2012b).

### 3.3 LROC NAC Reflectance

The reflectance of the WCK is indistinguishable from that of much of the surrounding mare due to the overprinting of immature ejecta from Proclus crater (Figure 1b). We examined high‐resolution NAC images of the WCK and surroundings to search for regions that have been less affected by the Proclus impact event and may be more representative of the WCK itself. One region of the WCK that appears to lie between two portions of Proclus rays has a photometrically normalized reflectance of 0.065 ± 0.005, compared to 0.056 ± 0.004 for a nearby mare region that lies between ray segments. The lowest reflectance materials on the WCK are associated with the ejecta of small impact craters (Figure 2b). The reflectance of these regions is on average 0.062 ± 0.001, and the craters with low reflectance are several tens to a few hundreds of meters in diameter. Assuming a maximum depth of excavation of 10% of crater diameter, the low reflectance material originated up to a few tens of meters below the WCK's surface.

### 3.4 Composition: M^3^ Spectral Reflectance and Diviner CF Interpretation

Using M^3^ absorption band centers and depths, and Diviner CF values from across the Crisium region, three primary local lithologies are identified. The distribution of these materials is seen in the parameter maps corresponding to pyroxene abundance and composition in Figures 4a and 4b, and 5a and 5b. Example spectra of each material, along with quantitative estimates of absorption band depth and center, are provided in Figures 4c and 4d, and 5c and 5d. CF values across the Crisium region (Figure 6) are within the typical range previously observed by Diviner (e.g., Greenhagen et al., 2010) and, compared to M^3^, are best utilized to interpret the relative amount of feldspathic mixing between norite units, such as those found in the central peak of Yerkes crater. The properties of these materials are as follows:

1. Feldspathic Materials: These materials are identified based on their high albedo, absence of 1‐ and 2‐μm absorption bands, and their low CF values. From M^3^, feldspathic materials are observed primarily in massifs corresponding and adjacent to the inner ring of Crisium. Significant volumes of feldspathic materials were also excavated by Yerkes E in western Crisium. Yerkes E Crater formed on a topographic high, possibly an isolated ring massif, which could be the slumped crustal blocks transported into the interior during the modification stage of the Crisium impact event (Spectra 4 and 5; Figures 4 and 5). Given the limited compositional variation within lunar feldspathic materials (Donaldson Hanna et al., 2014), most of the observed CF value variations are caused by darkening effects of space weathering (Lucey et al., 2009). The strongest feldspathic signals are correlated with fresher craters, such as Proclus and Yerkes E, or on steeper slopes in the feldspathic materials found in massifs within and adjacent to the inner ring of Crisium.
2. Mare Basalts: These materials are identified based on their low albedo, strong 1‐ and 2‐μm absorption bands, relatively long wavelength 1‐ and 2‐μm absorption band centers, and their high CF values. Mare basalts are volcanically emplaced and contain abundant Ca,Fe‐rich pyroxenes and are pervasive throughout the Crisium interior (Spectra 6–9). From Diviner, the regolith developed on the mare basalts in western Crisium is intermingled with significant amounts of feldspathic materials from the ejecta of Proclus and other craters that decreases CF values and makes discerning mature highland materials and well‐mixed mare basalts difficult with CF values alone.
3. Norites: These materials exhibit low‐intermediate albedo, strong absorption band depths, relatively short wavelength absorption band centers indicating Mg‐rich, low‐Ca pyroxenes, and high CF values (within the typical range of mare basalts). Around our study sites in Crisium, norites are most readily observed in the walls, rim, ejecta, and central peak of Yerkes Crater.

#### 3.4.1 WCK and Yerkes Crater Central Peak

M^3^ parameter maps of the WCK region capture the mineralogical properties of the kipuka in relation to other local lithologies (Figures 4a and 4b). The 1‐μm band depth map (Figure 4a), which is sensitive to pyroxene abundance, shows that the WCK exhibits a lower pyroxene abundance than the surrounding mare floor and nearby noritic crater structures, but a higher pyroxene abundance than the feldspathic massifs in the region.

The map of the 1‐μm band center (Figure 4b) shows that regions of the kipuka adjacent to the mare floor exhibit mare‐like pyroxene compositions (Ca,Fe‐rich), suggesting lateral mixing between kipuka and mare floor materials (consistent with processes described by Li and Mustard (2000)). The remainder of the kipuka exhibits relatively short wavelength band centers, suggesting nonmare, more Mg‐rich pyroxenes. The most distinctive pyroxene compositions across the kipuka are associated with the superposed impact crater Yerkes V (whose ejecta dominates the northeastern portion of the kipuka) and the central fracture.

Several spectra (1–3; Figure 4) were obtained and analyzed from these features to characterize their mineralogical properties in more detail. The central fracture (Spectrum 1) exhibits relatively long‐wavelength absorption bands (Ca,Fe‐rich), compared to the rest of the kipuka. These band centers are consistent with a basaltic composition, although the pyroxene abundance is lower than mare basalts elsewhere in Crisium (based on band depths). Yerkes V Crater exhibits a heterogeneous composition: although most of the crater wall and ejecta exhibit relatively short‐wavelength absorption bands (Spectrum 2) indicative of Mg‐bearing pyroxenes, there is a low reflectance streak on the eastern crater wall that is associated with much longer‐wavelength absorption bands consistent with a basaltic composition (Spectrum 3). We hypothesize that other WCK craters with low reflectance ejecta have similar compositions as the Yerkes V dark streak, but they are below the spatial resolution of M^3^. Nearby, weak absorption bands and high albedos suggest locally low pyroxene abundance (Spectrum 2). The central fracture and a dark streak near Yerkes V (Spectra 1 and 3) exhibit much lower albedos with somewhat stronger absorption bands, suggesting relatively high pyroxene abundance (but not as high as mare basalts; Spectra 6–9).

The central peak of nearby Yerkes Crater appears to exhibit a homogeneous noritic composition (Figure 4 and supporting information Figure S1). Heterogeneity across the central peak appears mostly related to downslope soil development and mixing with local materials at the base of the peak, a compositional pattern, which has been previously noted for noritic central peaks within the South Pole‐Aitken Basin (Moriarty et al., 2013). Representative materials from the central peak (Figure 4; Spectrum 10) exhibit short‐wavelength band centers and relatively deep absorption bands, indicative of abundant high‐Mg, low‐Ca pyroxenes.

As discussed in section 1.2.2, Yerkes central peak materials are likely uplifted from within the Crisium melt sheet. Proximal ejecta from the Yerkes‐forming impact were excavated from shallower depths of 1.5–3.5 km, estimated based on a range of reasonable scaling rules (0.05 times the final crater diameter; Osinski et al., 2013; 0.1 times the final crater diameter; Cintala & Grieve, 1998). From the M^3^ parameter maps (Figure 4), these ejecta also appear highly noritic (Figure 4 and supporting information Figure S1). This suggests that the melt sheet is homogeneous at this location over depths of several kilometers.

Prominent rays from Proclus Crater are superposed on the WCK region, suggesting interpretive caution regarding local and regional geology. To constrain the possible effects of these rays on compositional interpretations, spectra, band centers, and band depths from two mare soils (one on‐ray, one off‐ray) are included in Figure 4. Spectrum 9 was collected from a mare soil affected by a ray from Proclus, while Spectrum 8 was collected from a mare soil that does not exhibit signs of ray emplacement. Spectral interpretation of these soils (Figure 4c) confirms that the mare soil with the ray (Spectrum 9) exhibits a higher albedo than the off‐ray mare soil (Spectrum 10). However, the ray appears to have had only negligible effects on the properties of the 1‐ and 2‐μm absorption bands, as differences between the band depths and band centers of Spectra 8 and 9 are small (Figures 4d and 4e). From this analysis, it is reasonable to assume that the presence of Proclus ray material primarily affects albedo but does not significantly affect compositional interpretations of pyroxene abundance and composition based on the depths and centers of the 1‐ and 2‐μm absorption bands. As shown in Figure 6, the kipukas of the WCK generally have shorter‐wavelength CF positions than the surrounding mare basalts. The difference in CF position persists despite a significant shortward shift in CF positions for the mare basalts in western Crisium caused by the feldspathic Proclus ejecta. The central peak of Yerkes crater also has a CF position that is at shorter wavelengths than the mare basalts that have filled in the floor of the crater. However, the CF position for the Yerkes central peak is at a substantially longer wavelength than the WCK. Based on data from Diviner in combination with M^3^, the material exposed in the central peak and crater rim of Yerkes crater has an overall higher abundance of pyroxene than found in the noritic materials of the WCK. The CF values within Yerkes central peak support the M^3^‐based interpretation of a higher pyroxene abundance.

#### 3.4.2 Northern Archipelago and Other Kipukas

In contrast to the WCK and Yerkes Crater, the Northern Archipelago does not exhibit a particularly distinctive mineralogical signature (Figure 5). While substantially more feldspathic than the surrounding mare floor, the mafic component present across the Archipelago appears to reflect the general compositional gradient across the transition from Mare Crisium to the feldspathic inner ring massifs, likely indicative of impact mixing of the two lithologies. From band center measurements, contamination by mare materials is also observed in the relatively feldspathic massifs in the region.

While M^3^ spectra suggest that there is a modest gradient between feldspathic and basaltic units in the Northern Archipelago, the CF position boundaries are sharper, though still showing a gradient. Fresh craters across the inner ring massifs show strong feldspathic signatures while all of the kipukas have more intermediate CF values (Figure 6).

Though not explored in detail, the remaining candidate impact melt exposures identified by Spudis and Sliz (2017) (that is, north of the WCK and in eastern Crisium) are similar in morphology, setting, and compositional properties to the Northern Archipelago.

### 3.5 Radar Analysis

The surface and accompanying fractures of the WCK are identifiable in both the S‐ and P‐band radar coverage of western Crisium (Figure 7). Unfortunately, due to the small scale of the Northern Archipelago (diameters of <10 km across) relative to the radar resolution (80–400 m per pixel), it is not possible to distinguish distinct radar properties associated with the individual kipukas and so our radar analysis is restricted to the WCK.

There is no evidence of the Proclus ejecta and associated rays within either the same sense (SC) and CPR radar data sets, suggesting that the ejecta material is too fine/thin to be observed by the radar wavelengths employed. Within both SC radar data sets, the backscatter originating from the surface/near‐surface of the WCK is lower relative to that of the surrounding mare (it appears darker in the radar images), though the distinction is more apparent within the P‐band image (see arrows in Figure 7). SC radar backscatter is a product of scattering from blocky material present on the surface, suspended within the regolith, and (where the radar penetration is sufficient) situated at the base of the regolith. The difference in the relative return between the two wavelengths suggests that there is an absence of scattering from subsurface rock populations buried beyond the probing depth of the S‐band signal (~<5 m). The exaggerated lower P‐band backscatter return could therefore imply that: (1) The regolith is sufficiently thick over the WCK to reduce the contribution of basal echoes, (2) the WCK regolith attenuates the radar signal to a greater extent than that of the surrounding mare, or (3) a combination of the two.

Radar loss studies of the lunar mare have established that titanium content has the largest influence on signal attenuation (Campbell et al., 2014; Morgan et al., 2016). Based on the compositional assessment of the WCK from the other data sets discussed above, it is unlikely that the loss properties are sufficient to result in the reduced P‐band backscatter. We, therefore, favor the assessment that the WCK regolith is thicker, consistent with being older than the surrounding mare.

## 4 Discussion

### 4.1 WCK Morphometry and Composition

Here, we consider the formation mechanism of the WCK, pulling from its morphometry and composition. Morphologically, the WCK is a fractured dome (Figure 2) and seems to resemble fractured domes in floor‐fractured craters. The WCK's fracture slopes (Figure 8) are essentially the same as for the shallow slopes of fractures (~15°) on crater floors, which are unlikely to be normal faults given their shallow slopes and overall appearance, though some fractures may have shallowed with age due to down slope regolith creep. The presence of these fractures suggests that the fracture‐forming mechanism of the WCK could be similar to the magmatic uplift mechanism for floor‐fractured craters and distinct from the mechanism responsible for impact melt fracturing. The location of the WCK between the inner depression and inner ring of Crisium suggests that this material is a mixture of feldspathic target material, proximal ejecta, and impact melt/breccia produced during basin formation and evolution. Similar mixtures are observed within the “Heterogeneous Annulus” of the South Pole‐Aitken Basin (Moriarty & Pieters, 2018).

Compositionally, the WCK exhibits a range of pyroxenes, including the Mg‐rich pyroxene seen in the norite of Yerkes' central peak (Figure 4; Spectrum 10), but the WCK's pyroxene abundance is reduced to ~10% or less, suggesting dilution from feldspathic mixing. Craters with low reflectance ejecta (Figures 2 and 4; section 3.4.1.) that superpose the WCK exhibit mare‐like, Ca,Fe‐rich pyroxenes; these could be from exposed dike gabbro or excavated mare basalts.

While the WCK contains Crisium impact melt, it is diluted. Morphologically, the evidence is equivocal regarding a floor‐fractured crater‐like magmatic uplift process or a fractured impact melt process. If the former, we may have identified this process operating outside a comparably sized crater for the first time.

### 4.2 Sequence of Events for Western Crisium

**Figure 9:**
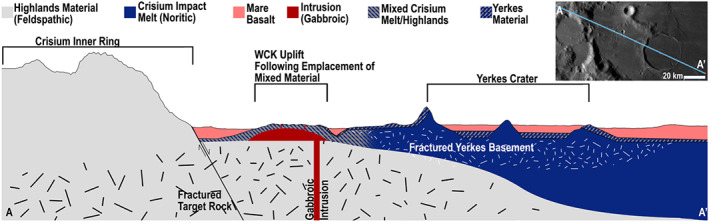
A qualitative, schematic cross section (~140 km long) along Crisium's inner massif ring, mare floor, the WCK, and Yerkes Crater. The surface topography was extracted from the 100 m per pixel global LOLA DEM in QuickMap with a surface vertical exaggeration of 5 times, but no scale is implied by the units at depth. Crisium's and Orientale's near‐identical sizes permit placing Crisium's melt sheet extents near where they are thought to be for Orientale (Neumann et al., 2015; Zuber et al., 2016). We interpret the Yerkes‐forming impact to have hit near the edge of the Crisium melt sheet, thereby creating a reprocessed melt sheet, breccia lens, and ejecta facies (“Yerkes Material”). Ejecta, slump facies, and some Crisium melt form the “Mixed Melt/Highlands” material. The Gabbroic Intrusion subsequently intruded and uplifted the floor of Crisium, emplacing dikes within the uplift. The entire area was then subsequently flooded by embaying mare basaltic volcanism. Impacts on the WCK exposed the dark, gabbroic dikes and the northwestern portion of the cross section was overprinted by ejecta from Proclus Crater.

How does Crisium's present‐day geomorphology result from past geologic events? We suggest the following model for the history of western Mare Crisium (Figure 9). First, an impactor excavated the Crisium basin, resulting in an interior with a 6‐ to 15‐km‐thick impact melt sheet. Owing to the thin crust (Wieczorek et al., 2013), this impact melt included lower crustal and possibly mantle material, resulting in a noritic composition; this stands in contrast to the Spudis and Sliz (2017) assumption of a highlands composition for the melt. Through ballistic emplacement of ejecta, slumping and translation during the basin modification stage, soil development, and impact‐driven mixing, feldspathic crustal material derived largely from the nearby massifs mixed with the noritic impact melt and ejecta. Second, the Yerkes impact event occurred near the margin of the Crisium melt sheet, exposing noritic Crisium impact melt within its rim and central peak complex. Third, between Yerkes and the feldspathic inner ring massifs, a dike‐fed laccolith locally domed and cracked the impact melt‐bearing mixed material of the floor of the Crisium basin in the manner of floor‐fractured craters (Jozwiak et al., 2015), forming the region that ultimately became the WCK. Along with the uplift, smaller gabbroic dikes may have propagated upward from the laccolith but stalled at depth. Fourth, floods of mare basalt partially inundated and embayed the lowest portions of the Crisium floor, leaving only the WCK, high‐standing feldspathic massifs, and portions of Yerkes Crater exposed. Fifth, an impact formed Yerkes V Crater, exposing both Crisium impact melt‐bearing basin floor materials as well as mare basalt or dike gabbro, leading to the low reflectance ejecta streak to the crater's east. Smaller impacts excavated dike gabbro, leading to the numerous craters with low reflectance ejecta. Sixth, rays of feldspathic ejecta were emplaced across western Crisium by Proclus Crater.

From this sequence of events, we thus interpret the WCK to contain relatively small amounts of datable impact melt from the Crisium‐forming impact and to also be heavily modified by impact‐driven mixing, intrusive plutonism, and mass wasting from the nearby massifs. Proximal ejecta from Yerkes Crater, which extends roughly a quarter crater radius from the rim, exhibits a relatively low pyroxene abundance, but the dominant pyroxene composition is Mg‐rich rather than the Ca,Fe‐rich pyroxene composition of mare basalts. This composition is consistent with a provenance from the lower crust or upper mantle, as expected for an area found to have an extremely thin (or absent) crust (Wieczorek et al., 2013). The central peak of Yerkes Crater likewise contains Mg‐rich pyroxene, but in much higher abundance than found in the ejecta and therefore likely exposes the most accessible and pristine impact melt that remains from the formation of Crisium.

### 4.3 Interpretation of the Northern Archipelago

**Figure 10:**
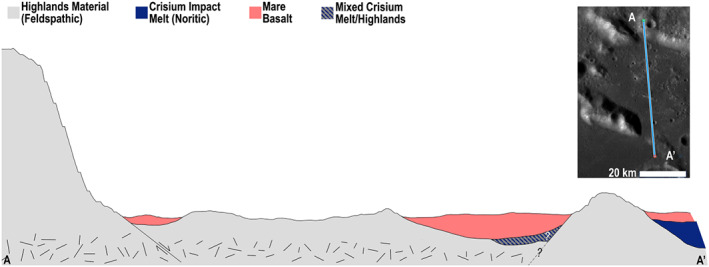
Same sense as Figure 8, but for the Northern Archipelago, ~60 km long. We interpret the kipukas to be related to the massifs, perhaps as slump blocks. The main impact melt sheet is interpreted to be southward (basinward) of the southern ring massif with perhaps some outward splash. Thus, we interpret the Northern Archipelago to not be a good candidate for sampling Crisium impact melt.

The Northern Archipelago is composed of high‐standing hills embayed by mare basalts located between segments of the inner basin rim. M^3^ spectra show most of the kipukas are strongly feldspathic, with lower pyroxene abundance than the mare but with mare‐like pyroxene composition (Ca,Fe rich). Additionally, the Archipelago shows a southward (basinward) gradient toward higher mafic content, matching the surrounding trend on the basin floor basalts. Thus, the kipukas could be slump blocks from the massifs that have likely experienced contamination from the surrounding mare via ejecta emplacement and regolith gardening. We suggest that after Crisium‐forming impact, blocks of massif material slumped into the valley and were subsequently embayed by mare basalts (Figure 10).

While we did not perform an in‐depth study of other circum‐Crisium kipukas originally identified by Spudis and Sliz (2017), our initial survey suggests they are similar to the northern Archipelago in terms of their morphology and feldspathic composition. Therefore, it is more likely that these kipukas are massif‐related rather than Crisium impact melt.

Kipukas other than the WCK and Northern Archipelago are consistent with crustal blocks embayed and contaminated to varying extents by subsequent mare emplacement and regolith gardening and development.

### 4.4 Sample Location and Exploration Concept

The peak and rim of Yerkes Crater are likely the purest outcrops of Crisium impact melt in these study regions, as evidenced by composition and estimated depth of origin compared to the expected vertical and lateral extent of the Crisium melt sheet. However, it would be advantageous to model the formation of Yerkes Crater to determine the likely temperatures and pressures this material experienced, the effect of elevated temperatures and pressures on the mineral assemblage of the Crisium impact melt sheet, and which isotopic systems may have been reset in the process. These impact melt materials in the central peaks may record the formation age of Crisium, even after being uplifted by Yerkes. These materials are likely only lightly shocked; the pressures experienced by Yerkes' central peak during uplift are likely less than 25 GPa (onset of partial melting; Johnson & Hörz, 2003) based on the observation of crystalline anorthosite in, and simulations of, the much larger Chicxulub Crater peak ring (Abrams et al., 1979; Baker et al., 2016). Zircons dated from the central uplift of Mistastin impact structure in Labrador, Canada, showed minimal perturbation due to the impact (Young et al., 2016), while feldspar in the central peak of the 23‐km‐wide Lappajärvi terrestrial crater was fully reset by the impact (Schmieder & Jourdan, 2013). Which of these scenarios, if any, are applicable to Yerkes Crater remains to be seen.

**Figure 11:**
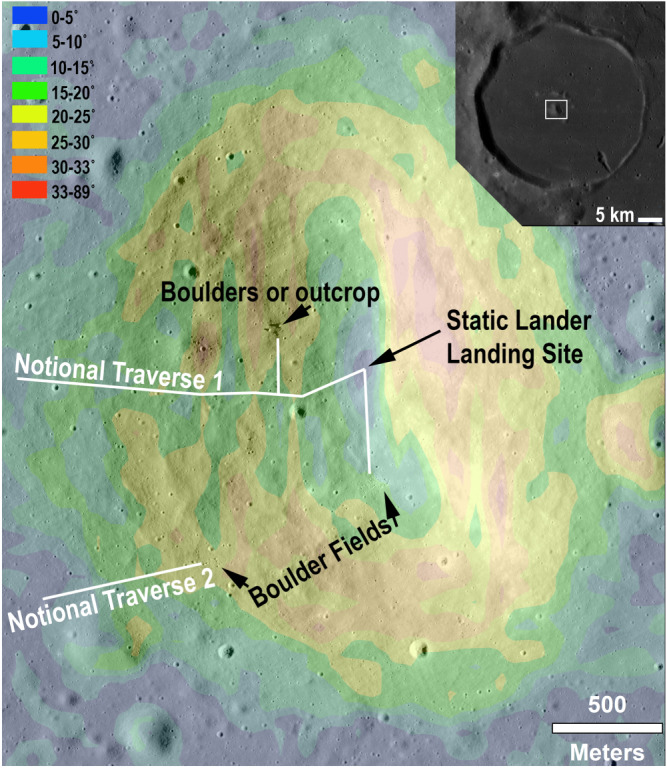
Exploration concept for sampling Crisium impact melt in the largest peak of the Yerkes Crater central peak complex (context shown, inset). Navigable slopes under 25° (yellow colors and cooler) would permit summiting the central peak of Yerkes Crater to reach the most pristine Crisium impact melt brought up from depth and to look for compositional gradients. The notional traverses (white lines) avoid the highest slopes. Landing a spacecraft with in situ dating capability on top of the peak would permit dating the formation of the Crisium basin and would avoid slopes but would require precision landing technology. Base image: LROC/NAC. Slope map derived from the LOLA‐Kaguya global DEM.

To sample impact melt, summitting the peak would not be necessary (Figure 11), although it could allow for additional unique investigations; for example, sampling compositions from different heights (corresponding different uplift depths) would allow for the investigation of compositional gradients in the initial impact melt sheet and therefore allow testing of the competing melt sheet differentiation hypotheses (e.g., Spudis et al., 2014; Vaugan et al., 2013). Additionally, determining a robust statistical age for the Crisium event would benefit by sampling a number of spatially separated bedrock locations, such as would be found along a floor‐to‐summit traverse. In order to sample the largest central peak of Yerkes, we assume a robotic, human, or human‐assisted robotic exploration architecture. If a static lander architecture were imposed, landing on top of the largest central peak, or at the base where material has been shed to lower elevations, would allow the lander to either date the material in situ or return it to Earth for more detailed laboratory analyses (e.g., Cohen et al., 2014, 2018; Ryder et al., 1989).

Precision landing within <25‐m accuracy is well within current lander technology, such as with optical terrain relative navigation (e.g., Criss et al., 2010; McGee et al., 2015). Our slope map, derived from an LROC DEM, shows a ~500 × 200‐m zone around the summit with slopes under 10° amenable to such a landing. There are several notable advantages to landing directly at the summit. One advantage is that it obviates the need to traverse up steep slopes and allows for a static lander. Another advantage is that it maximizes the chances that random regolith and rock grab samples are in situ: mass wasting from steeper slopes on the central peak would remove material and therefore constantly expose fresh material beneath; and the elevation and isolation of the summit minimizes the chance of foreign ejecta contamination. There are also disadvantages to direct‐summit landing with a static lander. One disadvantage is that it decreases the chances of sampling diverse locations to confirm a single age and to test for possible compositional gradients in the impact melt sheet (e.g., Vaugan et al., 2013). Surface mobility—whether by rovers or astronauts—opens up new concepts of operation, discussed below.

Surface mobility—regardless of summit or plains landing—enables collection of the most desirable geologic samples, such as outcrops or specific “float” rocks that can be confidently linked to a nearby (likely uphill) outcrop. Surface mobility would also allow for the acquisition of multiple high‐priority sampling targets. A mobile sampling and dating mission would benefit from multispectral microimaging (100 s μm/pixel) capabilities (Núñez et al., 2019) to optimize sample collection for either in situ analysis or Earth return. Though a robotic mission equipped with the proper onboard instrumentation could successfully return dateable samples, including astronaut‐geologists on the ground would greatly increase the efficiency and potential for the best possible sample suite to provide robust age and composition constraints for Crisium's impact melt. Field geologists on the central peak could locate submeter scale outcrops, easily move around to assess the local context and could test geologic hypotheses in seconds to minutes. Deciding the best and most representative suite of samples to collect—and documenting them before collection—are skills that human geologists bring to the field. Rock hammers, core tubes, field‐portable spectrometers, microimagers, cameras, and sample containers would make up at least part of the compliment of surface tools.

A human or purely robotic mission landing on the mare plains within Yerkes Crater would ideally climb at least part way up the central peak to access pristine Crisium impact melt, which is constantly reexposed by mass wasting. Figure 11 shows two notional traverses from the mare plains up the peak, with all slopes under 25°; this would be navigable on foot, with an uncrewed robotic rover, and/or with a crewed rover, for example, the Space Exploration Vehicle (Garry & Bleacher, 2010). Several boulder fields near the traverse would also provide a means of sampling stratigraphically higher lithologies transported downslope via mass wasting.

## 5 Conclusions

Dating the formation time of large lunar basins from impact melts is a driving goal in investigating the early geologic, environmental, and biogenetic history of the Earth‐Moon system. Toward that end, we remapped kipukas initially identified as Crisium impact melt facies by Spudis and Sliz (2017) and characterized their regional geomorphology and composition, using these results to infer the subsurface stratigraphy and formation sequence.

In the process of characterizing putative impact melt, we have possibly identified an occurrence of a known intracrater magmatic process (Jozwiak et al., 2012, 2015, 2017) operating in a novel way: outside a crater. This occurrence may be manifested in the WCK, which we interpret as uplifted and mare‐embayed Crisium floor material. While the WCK likely contains impact melt from Crisium, it has been diluted by both feldspathic highlands and mare basaltic material. Other kipukas—notably the Northern Archipelago—have a highlands‐to‐mare compositional gradient reflective of local geology with Ca,Fe‐rich mafics. Interpretations of reflectance and emittance spectra suggest that any Crisium melt must be present in even lower levels than in the WCK and is thus not a strong exploration candidate to date Crisium.

In contrast to the kipukas, the nearby central peaks of Yerkes Crater are strongly noritic, and from crater scaling relationships would have been uplifted from depths within Crisium's impact melt sheet (~6–15 km). Expected central peak shock pressures below 25 GPa (Baker et al., 2016; Johnson & Hörz, 2003), together with evidence from terrestrial crater peaks (Morgan et al., 2016; Young et al., 2013, 2016), strongly suggest that Crisium impact melt uplifted in Yerkes' central peaks could record the age of the Crisium‐forming impact.

A landed mission—whether human or robotic—to the top or base of the central peak complex of Yerkes Crater could best address the question of the age of the Crisium impact event. In situ age determination (e.g., Cohen et al., 2014; Cohen et al., 2018) or dates derived from returned samples could constrain the crystallization age of this impact melt, providing important new information about the bombardment history of the Moon and Earth.

## Supporting information



Supporting Information S1
