# Supplementary material for: Impact Melt Facies in the Moon's Crisium Basin: Identifying, Characterizing, and Future Radiogenic Dating
Source: J Geophys Res Planets. 2020 Jan 5;125(1):e2019JE006024. doi: 10.1029/2019JE006024 (PMC7375055; doi:10.1029/2019JE006024)
Supplement: Supplementary file 1 — Supporting Information S1 [file JGRE-125-e2019JE006024-s001.pdf]

Journal of Geophysical Research - Planets

Supporting Information for

Impact Melt Facies in the Moon's Crisium Basin: Identifying, Characterizing, and Future Radiogenic Dating

K. D. Runyon[1], D. Moriarty[2], B. W. Denevi[1], B. T. Greenhagen[1], G. Morgan[3], K. E. Young[2], B. A. Cohen[2], C. H. van der Bogert[4], H. Hiesinger[4], and L. M. Jozwiak[1]

[1]Johns Hopkins University Applied Physics Laboratory.

[2]NASA Goddard Space Flight Center.

[3]Planetary Science Institute.

[4]Institut für Planetologie, University of Münster.

Corresponding author: Kirby Runyon (kirby.runyon@jhuapl.edu)

Contents of this file

Figures S1
Tables S1-S2

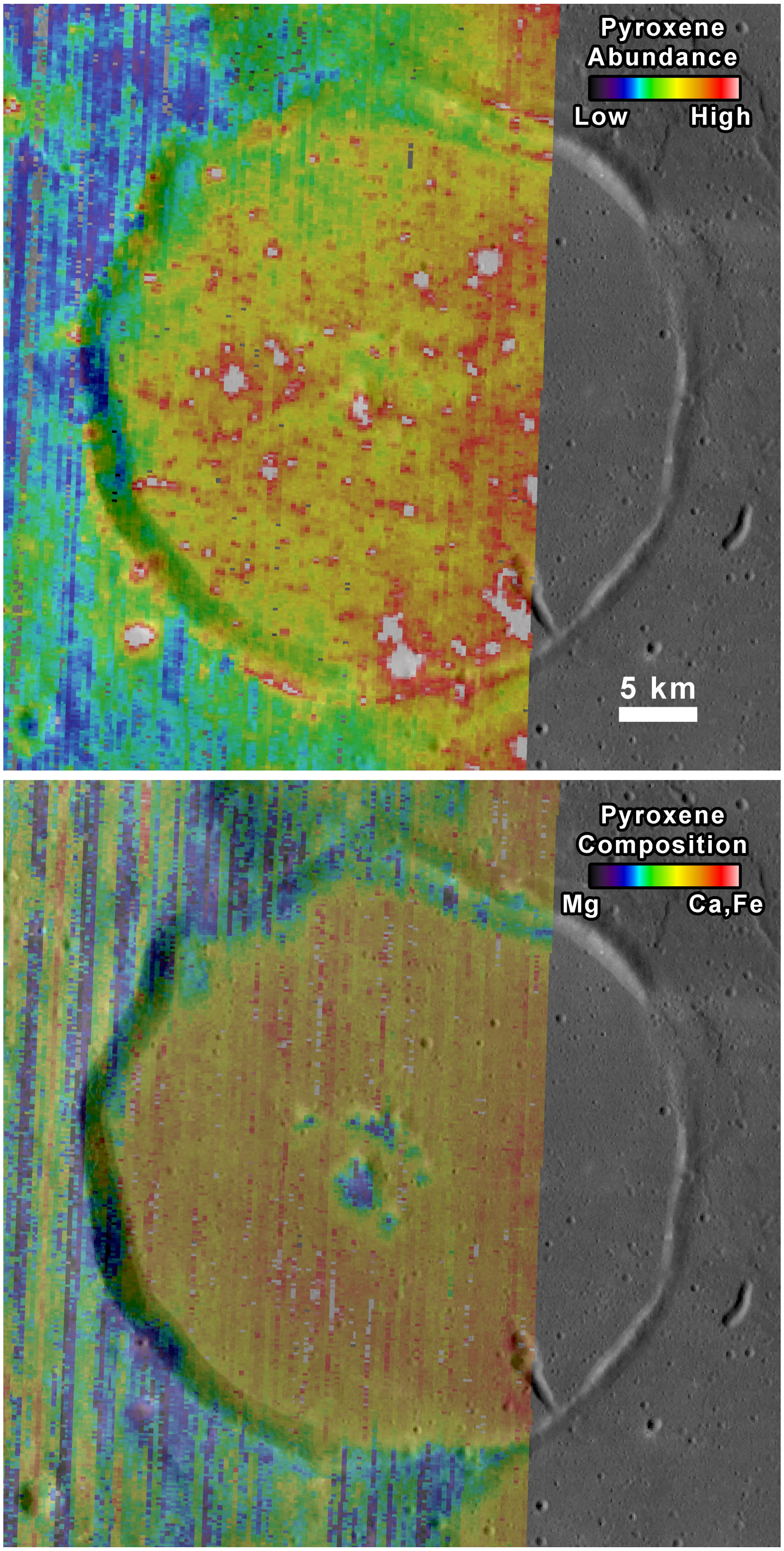

Figure S1. Same sense as Figures 4-5 but for Yerkes Crater. See text for results and discussion.

WCK Moderate Incidence
M1096165130LE
M1138575011LE
M1138575011RE
M1188024140LE
M1188031170LE
M1188031170RE
M1220974883LE
M134856157LE
WCK Low Incidence
M1113844229RE
M1116195394LE
M1131508919LE
M1220974883RE
M1240987762RE
M141933121LE
M141933121RE
M174942476RE
M187914267LE
M187914267RE
Northern Crisium Moderate Incidence
M104111232RE
M1098444270LE
M1123199851LE
M1123199851RE
M1142049056RE
M1199721182LE
M1199721182RE
M1230314770LE
M1233863715LE
M1233863715RE
M1236205631RE

Table S1. List of LROC NAC images used to analyze the Western Crisium Kipuka (WCK) and the Northern Archipelago.

Fracture Slope Measurements, Angles in Degrees				
	Max Slope	Min Slope	Slove (Averag)	Slope Standard Deviation
Atlas Floor-fractured Crater	34.3	2.5711	15.5	8.3
East Nectaris Floor Fractured Crater	20.0	9.6075	15.0	4.7
Western Crisium Kipuka	19.7	12.567	15.4	2.709682972
Southeast Imbrium Fractures	40.9	20.775	31.9	7.583209099
East Orientale, Non-Kipuka	38.9	20.609	26.9	7.070000539
South Orientale Melt Sheet	45.4	23.535	39.1	7.542842614

Table S2. Fracture slopes for various features discussed in the main text.
